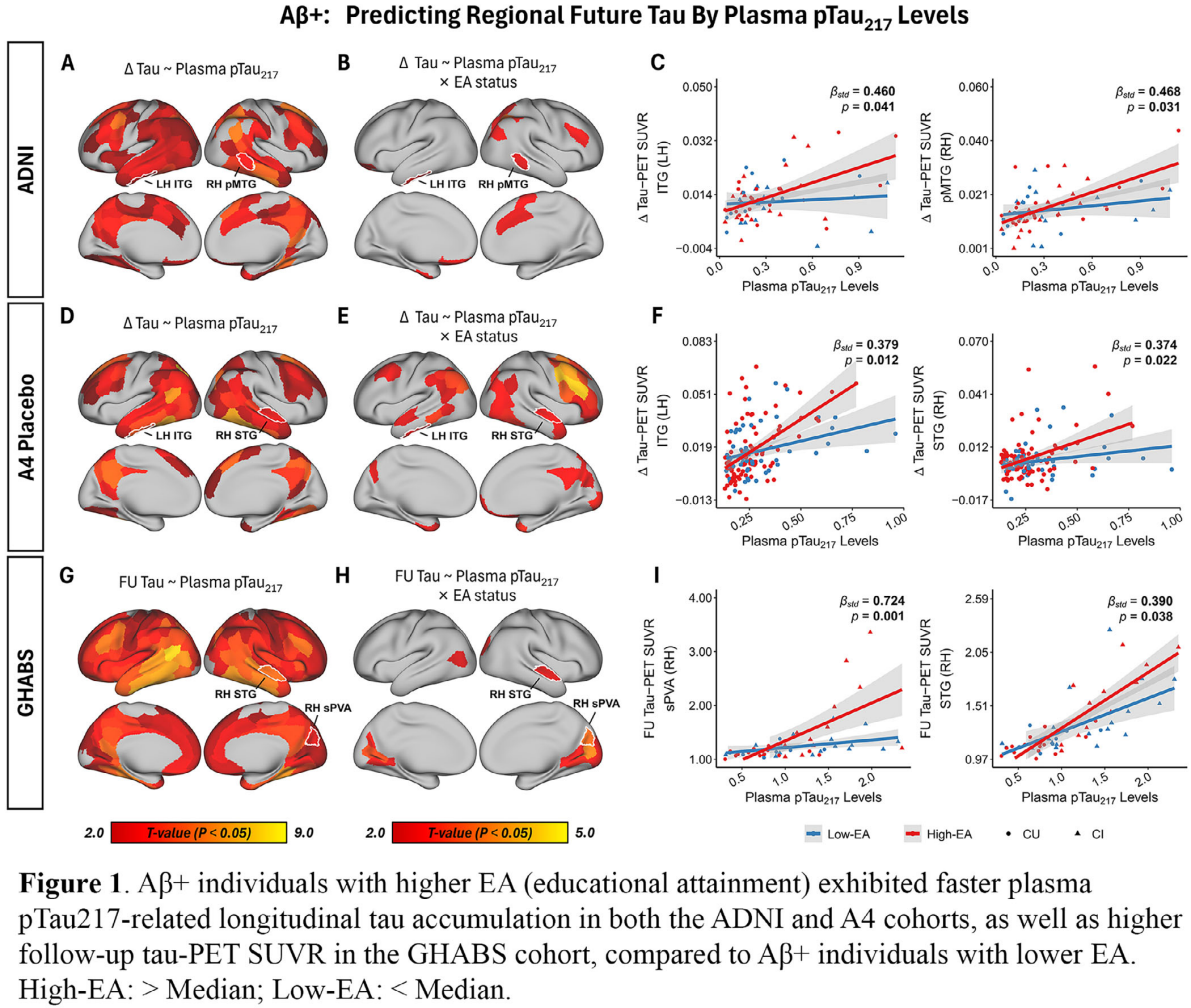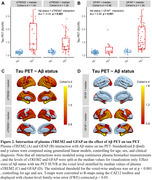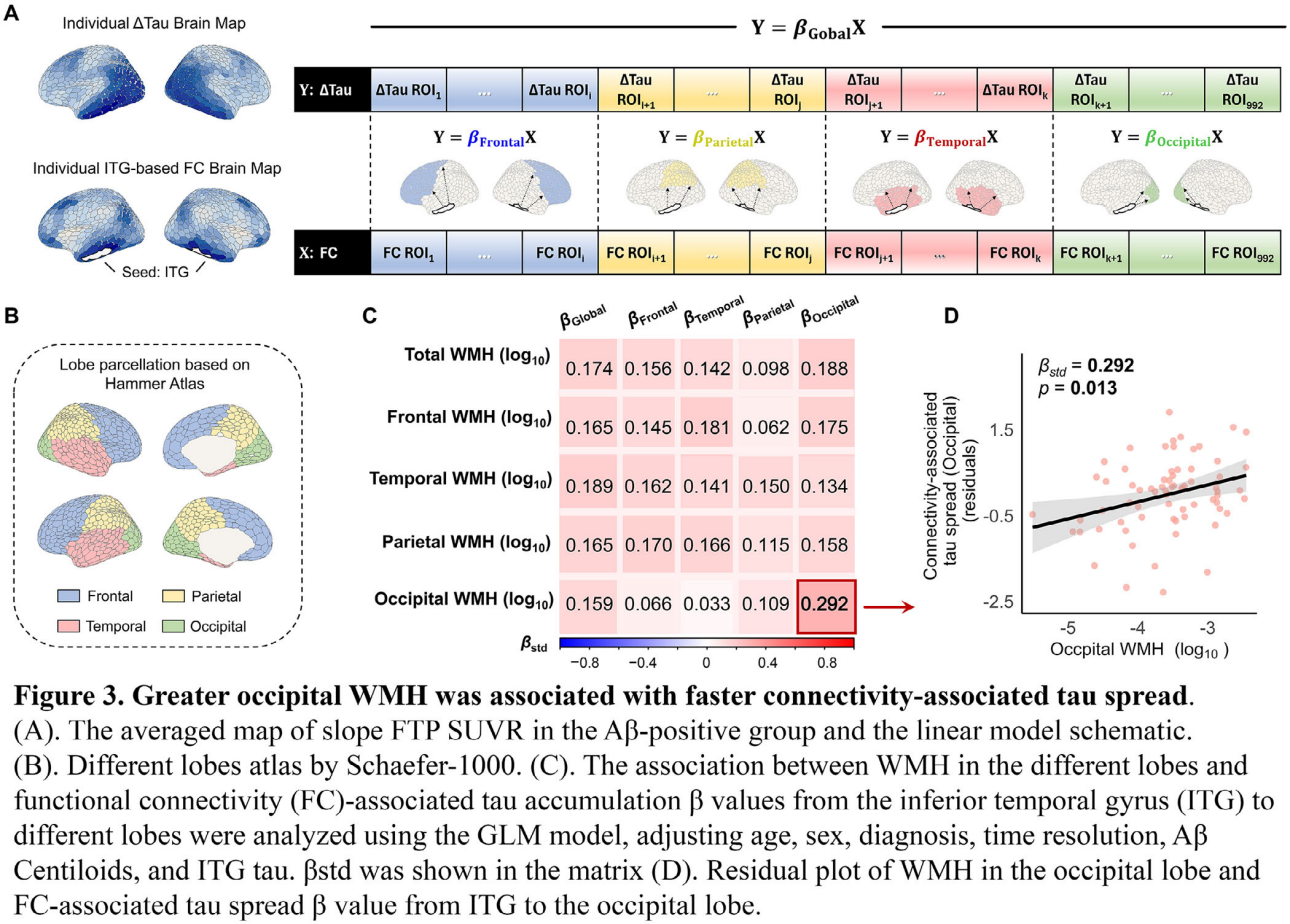# Unveiling Factors Contributing to Cortical Tau Tangle Accumulation and Spread Beyond β‐Amyloid in Alzheimer's Disease

**DOI:** 10.1002/alz70856_101561

**Published:** 2025-12-25

**Authors:** Tengfei Guo, Yue Cai, Jie Yang, Guoyu Lan

**Affiliations:** ^1^ Shenzhen Bay Laboratory, Shenzhen, Guangdong, China; ^2^ Institute of Biomedical Engineering, Shenzhen Bay Laboratory, Shenzhen, China; ^3^ Peking University Shenzhen Graduate School, Shenzhen, Guangdong, China; ^4^ Institute of Neurological and Psychiatric Disorders, Shenzhen Bay Laboratory, Shenzhen, Guangdong, China

## Abstract

**Background:**

β‐amyloid(Aβ) plaques and neurofibrillary tau tangles are key hallmarks of Alzheimer's disease (AD). Previous studies suggest that Aβ plaques are strongly linked with faster tau accumulation in the early AD stage. However, it is still not fully understood how other factors, such as educational attainment (EA), neuroinflammation, and vascular disease, contribute to cortical tau tangle aggregation and spread in AD.

**Method:**

To reveal how these factors affect cortical tau accumulation in AD, we analyzed tau PET imaging data from both the East Asian older population (Greater‐Bay‐Area Healthy Aging Brain Study, GHABS) and the Westen older population (ADNI and A4 studies). We investigated how EA status (Higher> Median Vs. < Median), plasma soluble triggering receptor expressed on myeloid cell 2 (sTREM2) and Glial fibrillary acidic protein (GFAP) concentration, and white matter hyperintensities (WMH) correlate with cortical tau accumulation or modulated the Aβ‐, entorhinal tau‐, and plasma phosphorylated tau (*p*‐Tau)‐related cortical tau aggregation.

**Result:**

Across the ADNI, A4 placebo, and GHABS cohorts, faster tau accumulation or higher follow‐up tau PET standardized uptake value ratio (SUVRs) in Aβ+ individuals with High‐EA were more strongly associated with higher global Aβ burden, entorhinal tau levels, and plasma *p*‐Tau217 levels (Figure 1), compared to those with Low‐EA. Higher plasma sTREM2 and GFAP were related to the weaker and stronger Aβ‐related tau accumulation, respectively (Figure 2). Furthermore, we found that higher WMH burden was associated with faster tau accumulation in the occipital lobe independent of Aβ pathology, which may be potentially explained by that greater occipital WMH burden linked to faster connectivity‐associated tau spread in the occipital lobe (Figure 3).

**Conclusion:**

These findings unveiled that longer education periods, lower plasma sTREM2 levels, higher plasma GFAP levels, and larger WMH burden are related to faster tau accumulation in AD in addition to Aβ pathology. This study emphasizes the importance of controlling these factors beyond Aβ to prevent tau aggregation in AD.